# Can takotsubo cardiomyopathy be diagnosed by autopsy? Report of a presumed case presenting as cardiac rupture

**DOI:** 10.1186/s12907-017-0045-0

**Published:** 2017-04-05

**Authors:** Andrew Mitchell, François Marquis

**Affiliations:** 1 Department of Anatomic Pathology and Cytology, Maisonneuve-Rosemont Hospital, 5415 Boulevard de L’Assomption, Montreal, Quebec H1T 2M4 Canada; 2 Departments of Medicine, Maisonneuve-Rosemont Hospital, 5415 Boulevard de L’Assomption, Montreal, Quebec H1T 2M4 Canada

**Keywords:** Takotsubo, Stress, Cardiomyopathy, Autopsy, Cardiac, Rupture

## Abstract

**Background:**

Takostsubo (stress) cardiomyopathy (TC) is a clinical syndrome featuring transient left ventricular dysfunction and wall-motion abnormalities, usually following emotional or physical stress. The diagnosis of TC depends on fulfillment of multiple clinical criteria. Although the pathogenesis has not been firmly established, myocardial cathecholamine toxicity is thought to represent a primary mechanism.

The vast majority of patients with TC survive. However, a rare cause of death in TC is myocardial rupture. All documented cases of rupture have followed known, recently diagnosed or suspected TC. However, in this report we propose that an initial diagnosis of TC with myocardial rupture can be made by autopsy when supported by a compelling clinical history and appropriate histologic changes in the myocardium.

**Case presentation:**

An 82 year-old female underwent elective craniotomy for a recently discovered craniopharyngioma. The surgery was uneventful; the initial postoperative course featured diabetes insipidus and delirium. With no prior warning, on the third postoperative day she was found unresponsive in bed. Two prolonged cardiopulmonary resuscitations were successful, however, during a third arrest maneuvers were stopped at the request of the family. An autopsy was conducted which revealed hemopericardium due to cardiac rupture. Coronary artery atherosclerosis, valve disease, and renal and extra-renal pheochromocytoma were absent. Microscopy of the myocardium showed a recent, localized, transmural myocardial infarction and diffuse changes (all four ventricles) typical of cathecholamine cardiomyopathy. The findings were considered compatible with TC with secondary myocardial rupture.

**Conclusion:**

An initial diagnosis of TC with myocardial rupture can be reasonably made by autopsy in the context of an appropriate clinical history and the presence of the characteristic microscopic features of cathecholamine excess in the myocardium.

## Background

Takostsubo (stress) cardiomyopathy (TC) is a well-recognized clinical syndrome featuring transient left ventricular dysfunction and wall-motion abnormalities, usually following emotional or physical stress [1]. Elderly women are typically affected. A summary of the Mayo Clinic criteria for diagnosis of the syndrome are: 1) transient abnormality of left ventricular wall motion beyond the territory of a single epicardial coronary artery, 2) absence of obstructive coronary coronary artery disease or angiographic evidence of acute plaque rupture, 3) presence of new ECG abnormalities or elevation in cardiac troponin levels, and 4) absence of pheochromocytoma and myocarditis [1, 2]. Concerning pathogenesis, neurogenically mediated coronary microvascular spasm and direct myocyte injury due to cathecholamine excess have been invoked [2, 3]. Although most patients survive, a recent study has shown that TC is associated with significant morbidity and mortality [2]. Cardiac rupture following TC is uncommon, with at least sixteen well-documented cases reported [4–8]. We report herein a presumed case of TC with cardiac rupture diagnosed by autopsy. The diagnosis is supported by the compelling clinical circumstances and the microscopic findings of cathecholamine cardiotoxicity.

## Case presentation

An 82 year old white female underwent elective craniotomy for resection of a recently discovered craniopharyngioma that had presented as diplopia. Her medical history included mild dyslipidemia, hypothyroidism and osteoporosis. Preoperative evaluation revealed an active lifestyle with no history of cardiac symptoms. A baseline ECG showed a left bundle branch block; no previous ECG was available for comparison. An endocrinological profile was carried out one week before surgery. The serum prolactin was elevated at 60 ng/mL (reference values: 5–24). The following were normal: serum IGF-1 64 ng/mL (33–185), serum TSH 1.86 mIU/L (0.3-4.2), FSH/LH ratio 4/0.8, serum cortisol 385 nmol/mol creatinine (289–35105). The serum sodium was 140 mmol/L (135–145). Other preoperative baseline laboratory values were normal.

The surgery was uneventful. Intravenous hydrocortisone was begun according to post-craniotomy protocol. The initial postoperative course featured diabetes insipidus and delirium. The sodium level rose to 148 accompanied by diuresis reaching 1 L per hour. Desmopressin was commenced; the sodium leveled descended to 139 on the second postoperative day. Of note, neither cardiac arrhythmia nor hemodynamic instability was documented.

On the third postoperative day she was found unresponsive in bed with agonal breathing. Prolonged cardiopulmonary resuscitation following standard ACLS protocol was promptly started. The patient was stabilized; an emergency head CT scan was performed which showed a small subdural hematoma. Then, approximately ninety minutes after the first episode, a second cardiac arrest occurred in the form of sudden pulseless ventricular tachycardia. The initial cardiac shock provided successful defibrillation. Point-of-care cardiac ultrasound showed a small pericardial effusion (less than 1 cm), normal left ventricular function, and a dilated and diffusely hypokinetic right ventricle. Shortly thereafter, sudden bradycardia with loss of cardiac output developed. During the following third resuscitation attempt cardiac massage and an external pacemaker resulted in no return of cardiac output. At this point the family requested that all maneuvers be stopped. Permission was granted to perform an autopsy.

### Autopsy findings

**Fig. 1:**
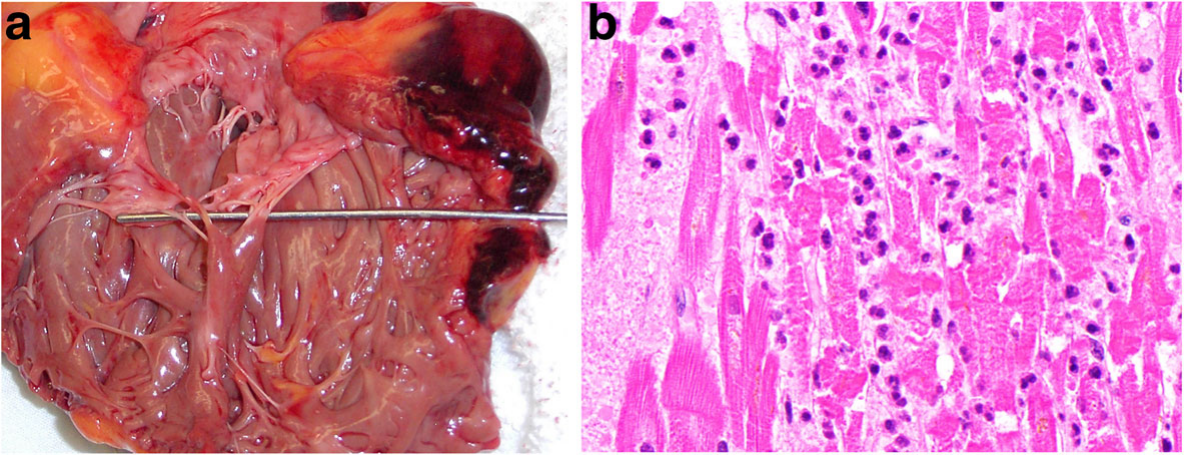
Macroscopic and microscopic features of the ruptured heart. **a** The probe passes through the rupture site at the base of the right ventricle. There is significant pericardial hemorrhage. The tricuspid valve is seen centrally. **b** High power photomicrograph of the infarct showing myocardial necrosis and a heavy polymorphonuclear infiltrate

A complete autopsy was performed. Hemopericardium (approximately one liter) was present. The heart weighed 360 g (predicted: 295 g, 95% confidence limits: 200–435 g) [9]. A slit-like rupture of the myocardium at the base of the right ventricule associated with epicardial hemorrhage was identified (Fig. 1a). Of note, no external cause for rupture such as a fractured rib was identified; the macroscopic features typical of a recent transmural myocardial infarction surrounding or near the rupture site were absent. Meticulous examination of the coronary arteries revealed absence of atheroma or acute thrombus. The cardiac valves were normal. The other organs were entirely normal. Adrenal and extra-adrenal pheochromocytoma were absent.

**Fig. 2:**
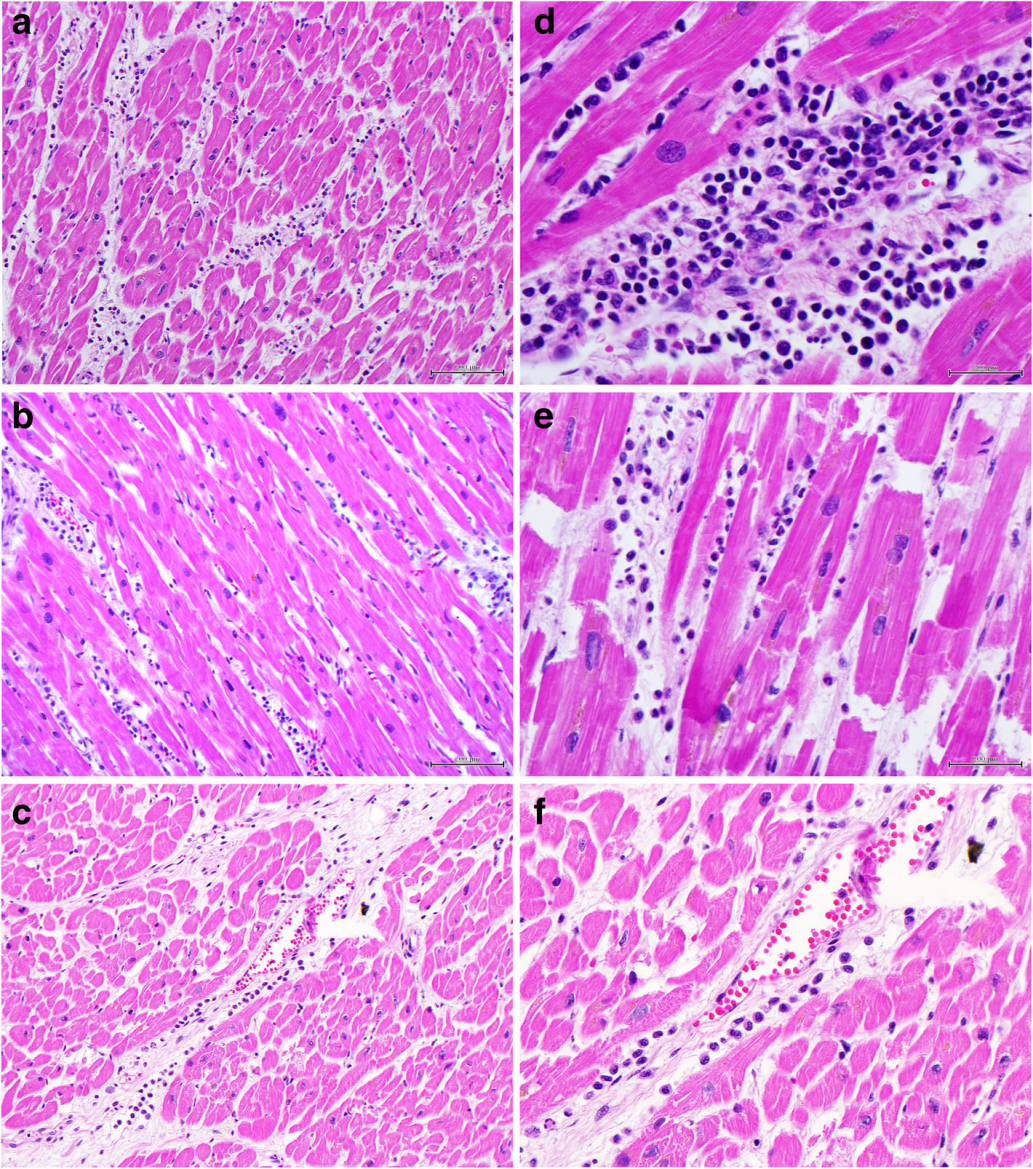
Microscopic features of the uninfarcted heart. **a**, **b**, **c** Medium power views showing interstitial edema with mononuclear cell infiltrates. **d**, **e**, **f** High power views of the mononuclear interstitial infiltrates. Rare polymorphonuclear leukocytes and eosinophils are present

Microscopic examination of sections from the rupture site showed epicardial hemorrhage and contraction band necrosis with heavy polymorphonuclear leukocyte infiltrates typical of a recent acute myocardial infarction, approximately 48–72 h [10] (Fig. 1b). The unusual site of the rupture, the absence of macroscopic features of an associated myocardial infarction, and the entire absence of coronary artery vascular disease, provoked a thorough examination of the rest of the myocardium. All thirty-four sections of grossly normal myocardium from the left and right ventricules revealed the following microscopic changes: areas of interstitial edema with patchy mononuclear cell infiltrates of lymphocytes and macrophages, and rare polymorphonuclear leukocytes, mast cells and eosinophils. These were found within the myocardium and also around intra-myocardial blood vessels (Fig. 2). There was no evidence of myocardial fibrosis. As these modifications involved the left and right ventricules diffusely, there was no correlation with the territory of an individual coronary artery. The findings were considered compatible with cathecholamine cardiomyopathy. The final autopsy diagnosis was of takotsubo cardiomyopathy with secondary myocardial rupture.

## Discussion

The pathogenesis of TC is considered to involve neurogenically mediated coronary microvascular spasm and direct myocyte injury due to cathecholamine excess [1, 2]. Evidence that cathecholamine excess is pathogenic derives from overlap of the histologic findings in myocardial biopsies of TC patients with those of myocardial biopsies and autopsy hearts of patients with conditions known to be associated with cathecholamine excess, including pheochromcytoma [11], acute emotional stress [3], subarachnoid hemorrhage and epilepsy [2]. Animal models of cathecholamine excess [11] have also demonstrated the same features. We believe such alternative etiologies of excess cathecholamines can be excluded in our patient. She was lifelong normotensive and there was no history of epilepsy. At autopsy the adrenal glands were normal and an extra-adrenal paraganglioma was not identified. (As there were no previous signs or symptoms suggesting cathecholamine excess, testing for 24 h urine cathecholamines had never been performed. Such testing is not routinely done prior to the type of surgery she underwent).

Descriptions of myocardial cathecholamine toxicity include the following: in an autopsy study of 26 patients with pheochromocytoma Van Vliet et al. found evidence of “active catecholamine myocarditis” in 58% of cases. “Disseminated focal lesions”, found in the myocardium of all four heart chambers, were associated with diffuse myocardial edema and were characterised by focal myocardial fiber degeneration and necrosis with “foci of inflammatory cells that were predominately histiocytes but also included plasma cells and occasionally polymorphonuclear leukocytes”. Lesions most often occurred around small blood vessels [11]. Wittstein et al. analysed the histologic findings in five patients undergoing myocardial biopsy following admission to a coronary care unit with chest pain or symptomatic heart failure following acute emotional stress. In four cases there were “interstitial infiltrates consisting primarily of mononuclear lymphocytes and macrophages and contraction bands without myocyte necrosis”. In the other case there was “an extensive inflammatory lymphocytic infiltrate and multiple foci of contraction-band myocyte necrosis” [3]. Akashi et al. observed “in most patients…interstitial infiltrates consisting primarily of mononuclear lymphocytes, leukocytes, and macrophages; myocardial fibrosis; and contraction bands with and without overt myocyte necrosis” [12]. In all instances, the findings differ significantly from the dense polymorphonuclear infiltrates and necrosis typical of acute myocardial infarction.

The differential diagnosis of the autopsy findings in our case includes “acute myocardial infarction-like syndrome with normal coronary arteries”, viral or post-viral cardiomyopathy, and a toxic or hypersensitivity drug reaction [13]. Although all of these entities may have myocardial mononuclear lymphocytic infiltrates, all typically present with signs and symptoms lasting several hours to days in the first entity to a few weeks to months in the others. Clinically, our patient had been afebrile, there had been no viral prodrome and she had not complained of dyspnea or chest pain before or after the surgery. In the months preceding surgery she had gone about her usual activities and traveled. Furthermore, evidence of left ventricular dilatation was absent both by the point-of-care cardiac ultrasound and at autopsy. We consider this lack of any clinical abnormalities preceding the fatal episode as being consistent with TC.

Cardiac rupture following TC is uncommon, with at least sixteen well-documented cases reported [4–8]. (Templin et al. in their recent review of 1750 patients with TC reported ventricular rupture in 0.2% of cases, but did not describe the pathologic findings [2]). In all cases the patients were female (age range 62–90), of which two survived. Autopsies were performed in seven cases; the others were diagnosed by catheterization or echocardiography. Rupture sites included the ventricular septum, left ventricular free wall (anterior, posterior, and apex), and the right ventricular wall. The histology of the rupture sites was reported in six cases. All showed the typical histologic findings of acute myocardial infarction: polymorphonuclear cell infiltrates and myocardial necrosis. However, in two cases “mononuclear lymphocytic infiltrations” [14] and “a mild mixed inflammatory cell infiltrate of lymphocytes and neutrophils” [6] were also described, raising in the latter “the possibility of an underlying resolving myocarditis”. Although the pathogenesis of myocardial infarction with rupture in TC is unknown, it is plausible that infarction may represent an ischemic epiphenomenon related to microvascular spasm [12].

Given the above observations, there appear to be circumstances in which the diagnosis of TC can reasonably be made post mortem. This is pertinent as a recent study found that TC is associated with significant morbidity and mortality: 7.1% of patients admitted to hospital suffer a major cardiac or cerebrovascular event within 30 days, the death rate from all causes is 5.6% per patient year, and the rate of major cardiac and cerebrovascular events is 9.9% per patient year [2]. Patients with TC may therefore come to autopsy more frequently than previously realized, and it is conceivable that the autopsy, albeit exceptionally, may be the first and only opportunity to make the diagnosis.

## Conclusions

We conclude, based on the findings in this case, that although TC is classically defined by clinical criteria it can be diagnosed at autopsy, as it has features that can be assessed by traditional pathologic evaluation, including distinctive diffuse mononuclear cell infiltrates, and the rare but well described occurrence of myocardial infarction with rupture. The clinical features of our case are those of a typical TC patient, and these, in correlation with the macroscopic and microscopic features of the heart, allow for a reasonably confident diagnosis of TC. However, it is important to note that our case represents a single preliminary observation. To confirm that autopsy diagnosis of TC is indeed feasible will require further reports of additional well documented cases.

Lastly, it is important to note the considerable value to the family and treating physicians of establishing by autopsy the cause of this upsetting sudden and unforeseen death.

## Abbreviations

ACLS: Advanced cardiac life support
ECG: Electrocardiogram
TC: Takotsubo cardiomyopathy
